# Chronic dietary supplementation with nicotinamide riboside reduces sleep need in the laboratory mouse

**DOI:** 10.1093/sleepadvances/zpad044

**Published:** 2023-12-20

**Authors:** Priyanka N Bushana, Michelle A Schmidt, Michael J Rempe, Barbara A Sorg, Jonathan P Wisor

**Affiliations:** Elson S. Floyd College of Medicine, Washington State University, Spokane, WA, USA; Elson S. Floyd College of Medicine, Washington State University, Spokane, WA, USA; Elson S. Floyd College of Medicine, Washington State University, Spokane, WA, USA; R.S. Dow Neuroscience Neurobiology Laboratories, Legacy Research Institute, Portland, OR, USA; Elson S. Floyd College of Medicine, Washington State University, Spokane, WA, USA

**Keywords:** antioxidants, dietary supplements, electroencephalography, nicotinamide adenine dinucleotide, nicotinamide riboside, oxidation-reduction, oxidative stress, reactive oxygen species

## Abstract

Non-rapid eye movement sleep (NREMS) is accompanied by a reduction in cerebral glucose utilization. Enabling this metabolic change may be a central function of sleep. Since the reduction in glucose metabolism is inevitably accompanied by deceleration of downstream oxidation/reduction reactions involving nicotinamide adenine dinucleotide (NAD), we hypothesized a role for NAD in regulating the homeostatic dynamics of sleep at the biochemical level. We applied dietary nicotinamide riboside (NR), a NAD precursor, in a protocol known to improve neurological outcome measures in mice. Long-term (6–10 weeks) dietary supplementation with NR reduced the time that mice spent in NREMS by 17 percent and accelerated the rate of discharge of sleep need according to a mathematical model of sleep homeostasis (Process S). These findings suggest that increasing redox capacity by increasing nicotinamide availability reduces sleep need and increases the cortical capacity for energetically demanding high-frequency oscillations. In turn, this work demonstrates the impact of redox substrates on cortical circuit properties related to fatigue and sleep drive, implicating redox reactions in the homeostatic dynamics of cortical network events across sleep–wake cycles.

**Statement of Significance**

Nicotinamide riboside (NR) is a biochemical precursor of nicotinamide adenine dinucleotide, a coenzyme critical for numerous oxidation-reduction reactions. Dietary exposure to NR reduced the amount of time that laboratory mice spent in non-rapid eye movement sleep (NREMS) by 17%. The equivalent in a human would be a reduction from 8 hours of NREMS to 6.6 hours. Mathematical modeling of the homeostatic regulation of sleep (“Process S”) indicated an accelerated rate of discharge of sleep need in mice subjected to dietary NR. Waking electroencephalographic markers were suggestive of reduced drowsiness with dietary NR even in the face of reduced time in NREMS. These data demonstrate a role for nicotinamide substrates in sleep homeostasis.

## Introduction

**Figure 1.**
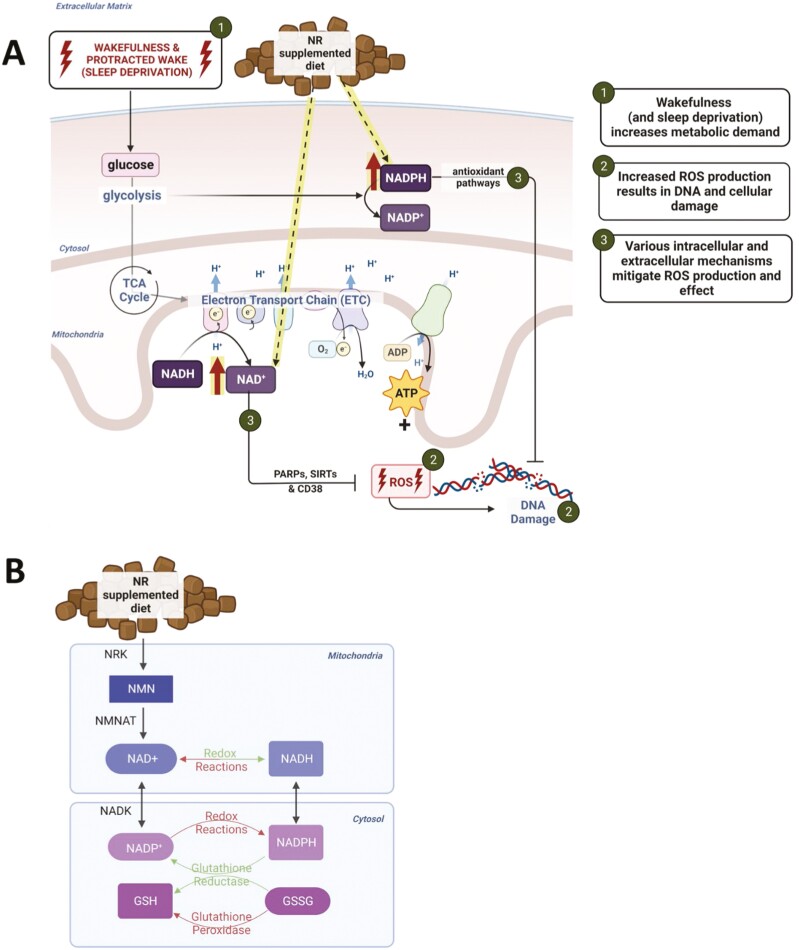
Supplementation of nicotinamide riboside (NR) increases NAD availability. (A) We expect that (1) wakefulness and prolonged wakefulness (caused by sleep deprivation) increases metabolic demand and glucose utilization. This creates an increased need for biochemical redox substrates through increased glycolysis (NADP/H) and cellular respiration (NAD/H). Both processes produce (2) increases in reactive oxygen species (ROS) and subsequent cellular damage (conceptualized here as DNA damage). (3) NAD(P)/H can mitigate ROS-related damage via various cellular antioxidant mechanisms, including through action of poly-ADP ribosyl polymerases (PARPs), sirtuins, and CD38. Our hypothesis suggests that increasing the availability of NAD(P)/H by NR supplementation further aids these antioxidant mechanisms in mitigating ROS-related damage produced by wakefulness. In the experiment discussed here, 12 control mice were placed on control chow, and 11 experimental mice were placed on diet supplemented with NR to increase the bioavailability of oxidized NAD^+^ in the brain. (B) demonstrates the biochemical pathways by which NR is converted to NAD^+^ via NR kinase (NRK)-driven phosphorylation to produce the biosynthetic intermediate nicotinamide mononucleotide (NMN). NMN is metabolized to NAD^+^ via NMN adenylyltransferases (NMNAT). NAD(P)^+^ and NAD(P)H serve as redox cofactors in reactions that produce ATP (adenosine triphosphate) [52]. Image created with BioRender.com.

The propensity for and depth of sleep increases in proportion to prior wake duration and declines in proportion to time spent asleep. Sleep homeostasis refers to this accumulation of sleep need during wake and its discharge during sleep. Changes in brain metabolism are among the most salient and robust of the constellation of physiological changes that characterize sleep. Brain glucose utilization drops precipitously in non-rapid eye movement sleep (NREMS). By some estimates, it is reduced by 50% during NREMS relative to wakefulness as indicated by positron emission tomography in human participants [1–3] and *post-mortem* assays in rodents [4]. Brain temperature and oxygen utilization are also reduced in NREMS relative to wakefulness. These profound metabolic changes have led to the speculation that downregulation of glucose metabolism is central to the functional utility and homeostatic dynamics of sleep [5, 6]. Among the biochemical correlates of sleep homeostasis is brain redox status: the brain accumulates oxidative stress during wake, and eliminates the biochemical markers of oxidative stress during sleep [7–9]. Glucose metabolism requires oxidation/reduction reactions involving nicotinamide adenine dinucleotide (NAD) [10–11], an obligatory cofactor for many redox reactions. The reduction in glucose metabolism that occurs in association with NREMS is thus accompanied by downregulation of oxidation/reduction reactions involving NAD. We thus hypothesized that a systemic manipulation of NAD might impact electroencephalographic (EEG) readouts of sleep homeostasis (visual summary schematized in Figure 1A).

The current study employed systemic administration of nicotinamide riboside (NR) to achieve this goal. The biochemical relationship of NR to NAD^+^ is schematized in Figure 1B. NR is converted to NAD^+^ by a two-step process that includes the synthesis of the intermediate nicotinamide mononucleotide (NMN). Oral administration of NR increases levels of NAD and related metabolites across the blood-brain barrier via the conversion to NAD^+^ or NADP^+^ and glutathione within cells of the brain parenchyma [12]. We hypothesized that the increased redox capacity generated by systemic administration of NR would provide more energy-transfer capacity and decelerate the accumulation of oxidative stress in the waking brain. If indeed oxidative stress contributes to sleep homeostasis, this manipulation would be expected to manifest as an alteration in the homeostatic regulation of sleep. We tested this hypothesis by measuring sleep state timing and modeling process S in animals subjected to dietary NR administration.

## Methods

### Ethical approval

This study was approved by the Institutional Animal Care and Use Committee of Washington State University and conducted in accordance with National Institutes of Health’s Guidelines for the Care and Use of Laboratory Animals. All efforts were made to minimize the number of animals used in the experiments and to reduce the amount of pain and suffering.

### Animals and surgery

**Figure 2.**
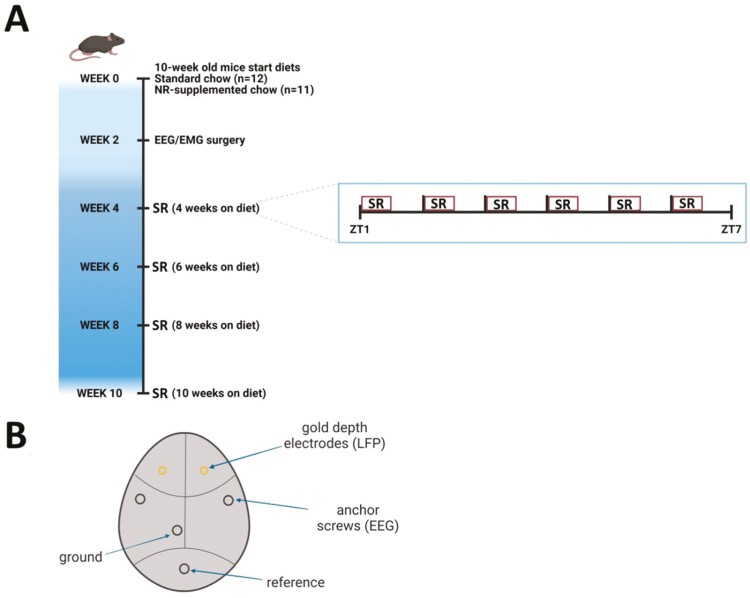
Experimental protocol. Full description is in the Methods section. The experimental design is diagrammed in (A) Briefly, adult C57BL6/J mice between 8 and 10 weeks of age were started on diets formulated by ResearchDiets Inc. Twelve mice were placed on ResearchDiets control chow (six males, six females) and 11 mice were placed on ResearchDiets control chow supplemented with NR (five males, six females). Two weeks after starting on diet, mice were implanted with gold depth electrodes (LFPs), EEGs, and nuchal EMGs, all soldered to a headmount connector (see (B) for implantation configuration). Every 2 weeks after EEG/EMG implantation, mice were subjected to a 24-hour baseline recording and immediately thereafter sleep restricted by gentle handling in a 30-minute SR on/30-minute off protocol, starting at ZT1. Mice were subsequently subjected to a 24-hour recovery sleep recording. The sleep restriction protocol is diagrammed in an inset in (A). sleep restriction (SR) periods are represented in red. 30-minute recovery sleep opportunities were interspersed between SR sessions from ZT1 to ZT7. Both images were created with BioRender.com.

C57BL/6J mice were singly housed in a vivarium which remained between 70° and 75°F at a relative humidity of 50%, on an LD 12:12 cycle. These experiments used two separate groups of mice in a between-participants independent measures design to assess the effects of NR supplementation on EEG and sleep-related parameters. In total, 33 mice (12 females, 11 males; all aged 10–12 weeks at the time of surgery) were anesthetized using isoflurane (5% induction; 1%–3% to maintain 0.5–1 Hz respiration rate) and placed in a stereotaxic frame. A 1-cm midline incision was made in the skin over the dorsal surface of the skull, and the skull was exposed to allow two holes, roughly 0.5 mm in diameter, to be drilled over predetermined coordinates targeting the medial prefrontal cortex (mPFC; A/P + 1.94; M/L ± 0.5; D/V −1.3). At this location, stainless-steel polyimide-insulated depth electrodes (Plastics One part #E363/1/SPC diameter: 0.25 mm) were implanted bilaterally for local field potential (LFP) measurements. Prior to surgery, depth electrodes were cut to 1.5 mm. Mice were additionally implanted with EEG and electromyographic (EMG) electrodes as diagrammed in Figure 2B. Two stainless-steel EEG screw electrodes were implanted over the parietal cortices and two EMG electrodes were implanted in the nuchal muscles. All electrodes were soldered to a six-pin headmount connector and secured to the skull with dental cement [13]. Buprenorphine sleep restriction (SR) (1.0 mg/kg) was administered once as a post-operative analgesic. After surgery, all mice were singly housed in a vivarium which remained between 70° and 75°F at a relative humidity of 50%, on an LD 12:12 cycle for 12–13 days during recovery from surgical procedures.

### Diet

At 10 weeks of age, half of the mice were switched from control chow (Harlan Teklad 2016) to an experimental (*n* = 5 males, 6 females) diet containing 3.33g NR/kg (ChromaDex), and half were maintained on a control (*n* = 6 males, 6 females) diet, substantially similar to the Teklad control chow (both diets formulated by Research Diets Inc, OpenSourceDiet) for 10 weeks. The experimental diet was formulated with the goal of achieving daily NR consumption of 400 mg/kg, which has been shown to impact neurological endpoints (specifically neurogenesis) in adult C57BL/6 mice [14], assuming that mice consume 5 g of chow daily. Mice were given ad libitum access to diet and water. Diet and animals were weighed twice weekly.

### Experimental design

At 14 weeks of age (i.e. 4 weeks on control or experimental diet), animals were connected to recording cables via headmount and re-housed. Mice were allowed to habituate to the data collection environment (a cylindrical acrylic plastic cage: 25 cm in diameter, 20 cm tall) and headmount tether overnight. After habituation, 24 hours of undisturbed baseline LFP/EEG and EMG recordings were collected starting at ZT1. Mice subsequently underwent SR for 6 half-hour intervals via automated sleep deprivation (Part #9000-K5-S, Pinnacle Technology, Inc.) supplemented by gentle handling during a 6-hour protocol, from ZT1 to ZT7. This protocol cycled 30-minute SR sessions with 30-minute recovery sleep opportunities (see Figure 2A), as described in Grønli et al, 2016 [15]. This SR model encourages the prevalence of quiet wakefulness (QW) during 30-minute recovery sleep episodes, increasing opportunities to study the effect of NR on the waking EEG. Recovery sleep was recorded for the 24 hours following the SR session. SR experiments were run in cohorts of six animals to maximize the capacity of the available recording equipment. Each cohort of animals was run in a repeated-measures design, undergoing three subsequent SR protocols at 16, 18, and 20 weeks of age (i.e. 6, 8, and 10 weeks on diet). This experimental design is schematized below (Figure 2A).

### Data collection and processing

Data from LFP, EEG, and EMG potentials was collected, extracted, and processed as described previously [13]. Briefly, LFP, EEG, and EMG signals from the head mount were fed through a PCB-based preamplifier (Part #8406-SL, Pinnacle Technology, Inc.) to a commutator (Part #8408, Pinnacle Technology, Inc.), which was read into a PC-based acquisition system (Pinnacle Technology, Inc.; Part #8401). Signals were further amplified 50-fold and sampled at 400 Hz.

LFP and EMG potentials were extracted using Pinnacle Technology Inc.’s Sirenia software. Files were extracted in European Data format (.edf) and contained data from two frontal LFPs and one nuchal EMG each. These sleep recording files were scored through the online computational tool SPINDLE (Sleep Phase Identification with Neural Networks for Domain-invariant Learning), in 4-second epochs [16]. The SPINDLE scoring method was validated against manually scored datasets in the published work [16] and in our laboratory, as described below.

EEG spectral data (.edfs) and vigilance state classifications (from.csv files) were processed together with the MATLAB computing language, as previously described [7]. EEG spectral data was separated into the following bands: δ (1–4 Hz), θ (5–8 Hz), α (9–12 Hz), β (15–35 Hz), γ (35–120 Hz). The gamma range was further subdivided into low γ (35–60 Hz), γ (60–90 Hz), and high γ (90–120 Hz). Wakefulness was subdivided into QW or active wakefulness (AW) using EMG peak-to-peak amplitude of all wake epochs across the entire recording. QW was defined as the 33rd percentile or less and AW the 66th percentile or higher of all wake EMG peak-to-peak amplitude values [15].

### SPINDLE scoring validation

**Figure 3.**
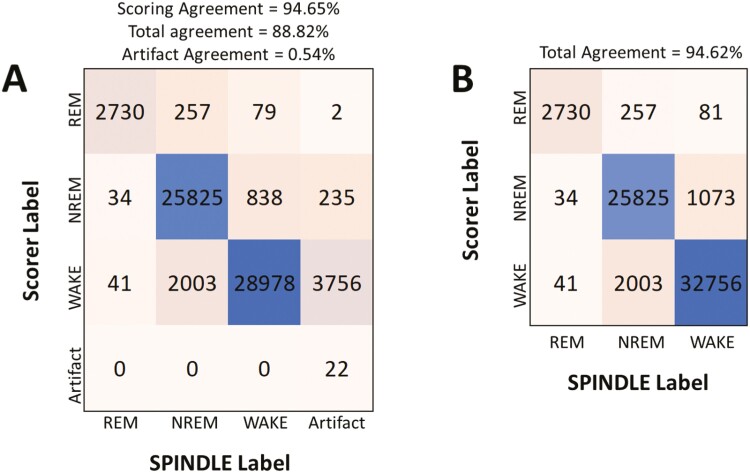
Confusion matrices derived from manual sleep scoring and SPINDLE scoring a set of three 24-hour LFP/EMG recording files. The number of epochs scored the same by SPINDLE and the manual scorer are shown at each intersection (diagonally placed numbers, from the top left to bottom right). Scoring agreement, total agreement, and artifact agreement were evaluated as described in Miladinović et al., 2019[15]. In Panel (A), the agreements were computed for non-artifact and artifact data separately (artifact agreement and scoring agreement), and again when taking all epochs into account (i.e. total agreement). Panel (B) shows the final version of our scoring process, where artifacts were collapsed with wake annotations.

In the original publication, SPINDLE scoring was validated by three independent labs, which achieved average agreement rates of 93%–99% [16]. This is generally on par with or better than average inter-individual agreement. Before using SPINDLE to autoscore our sleep state classifications, we assessed the average agreement rate between a subset of our own hand-scored data and SPINDLE scoring in files from NR-treated animals. The confusion matrix shown in Figure 3 demonstrates the agreement between manual and automated scoring across three 24-hour recordings. Our overall agreement rate was 88.82%, due to low agreement between hand-scoring and the SPINDLE algorithm in identifying epochs containing artifactual signal (0.54%). Artifacts were largely found during epochs deemed as wake by the manual scorer (Figure 3A); when artifact annotations were collapsed with wake annotations, the agreement rate increased to 94.62% (Figure 3B). All subsequent files were scored in this manner, with artifacts collapsed into wake (though excluded from EEG spectral analyses).

### Process S analysis

To quantify the dynamics of slow wave activity (SWA; delta: 1–4 Hz) during slow wave sleep (SWS or NREMS), we employed a previously published modeling framework [17, 18] following the Franken approach [19]. After finding all 5-minute overlapping segments that consisted of at least 90% SWS, we computed and plotted the median SWA during SWS. We then fit these data points with a simple homeostatic model (Process S) that rises exponentially during epochs classified as wakefulness or rapid eye movement sleep (REMS) and declines exponentially during SWS. The upper and lower asymptotes (UA, LA) were found (separately for each week’s recording) in a manner described by previous studies [19]: UA was chosen as 90% of the level of the distribution of SWA during SWS, and the lower asymptote, LA, was chosen as 10% of the level of the distribution of SWA during SWS. The asymptotes UA and LA were fixed constants within each recording but differed between recordings. There were only two free parameters in the model: the time constants for the rising (τ_i_) and falling (τ_d_) of Process S. τ_i_ and τ_d_ values were optimized for each recording using MATLAB’s *fminsearch* command, which identified the values that collectively minimize the residuals of actual delta power values relative to modeled values.

### Statistical analysis

Statistical analysis was performed with STATISTICA software (version 12.0, StatSoft, Tulsa, Oklahoma). Differences between means of sleep timings or EEG spectra were estimated by repeated-measures analysis of variance (RM ANOVA), with significance levels set to *α* ≤ 0.05. Independent variables assessed include treatment (diet), sex, week (4 vs. 6 vs. 8 vs. 10), and time of day. Data analysis was subjected to sigma-restricted parameterization and effective hypothesis decomposition methods by the software. Significant results were further investigated by Fisher’s LSD post hoc test.

## Results

### NR-diet-supplemented animals eat more chow but do not gain more weight than animals on control chow

Animal weights and food weights were assessed twice weekly during the experiment to monitor chow consumption. Over 10 weeks of monitoring, mice fed on the NR-supplemented diet exhibited a trend toward increased food intake relative to mice fed control chow (average daily NR-diet consumption = 3.60 ± 0.08 g; average daily control chow consumption = 3.37 ± 0.08 g; ANOVA results: F_1,19_ = 4.23, *p* = 0.054). There was no difference in weight change between the two groups. A two-way ANOVA additionally showed non-significant interactions between diet and sex for both daily food consumption and mouse weights. These data suggest that NR prevents weight gain. Generally, the literature does not show that NR consumption impacts weight gain, except when animals are fed high-fat diets. Several studies show that NR can attenuate weight gain on high-fat diets [20, 21] through alterations in body composition [22]. These differences in food consumption absent weight changes may be reflective of increased oxidative metabolism, decreased fat mass, and increases in sleeping metabolic rates [20, 22, 23].

### NR supplementation decreases overall time spent in NREMS during uninterrupted baseline activity

**Figure 4.**
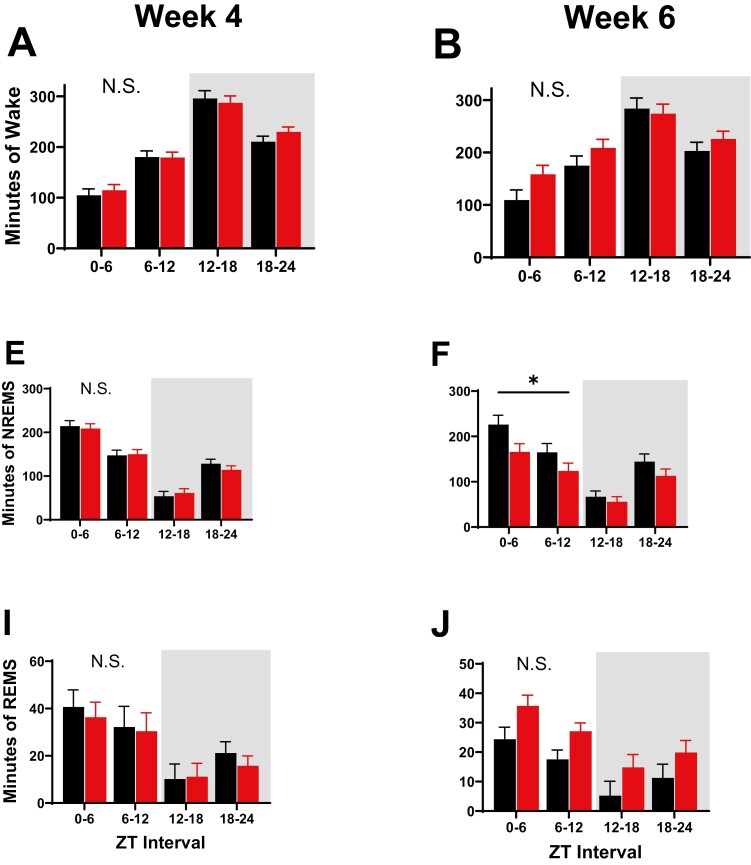
Sleep state classifications derived from SPINDLE-scoring of EEG/LFP and EMG during baseline (spontaneous sleep; SS) recordings. Bars represent the total number of minutes spent in each sleep state—wake (A–D), NREMS (E–F), and REMS (I–L)—during 6-hour bins when left undisturbed. Sleep state classifications were derived from 24-hour baseline recordings 4 (A, E, I) and 6 (B, F, J), weeks after starting experimental diets. No significant differences were noted in time spent in wake or REMS. Significant differences between NREMS sleep minutes in animals on control chow (black bars) and NR-supplemented diet (red bars) were indicated by asterisks between these two groups, where *p* < 0.05. These asterisks refer to main effect of treatment as assessed via RM ANOVA. Light and dark phases are denoted by the color of the background in each panel. Differences were not modulated by sex. Error bars signify standard error of the mean (SEM).

NAD^+^ levels oscillate with sleep–wake cycles [24]. Decreases in global NAD^+^ levels are associated with aging, metabolic disease, circadian disruption, and sleep–wake disruption [25]. Decreases in NAD^+^ levels can negatively impact sleep quality, and this finding has led to various investigations of NAD^+^ manipulations in the context of sleep [26, 27]. Until now, NR supplementation (to increase NAD^+^ availability) has not been assessed in the context of sleep efficiency. We hypothesized that chronic dietary NR supplementation would lead to changes in LFP and sleep architecture indicative of increased sleep efficiency. We assessed sleep state classifications during 24 hours of baseline undisturbed sleep in 6-hour bins at weeks 4, 6, 8, and 10 after starting experimental diets. No significant changes were observed in the time spent awake or in REMS across any of these time points (Figure 4, A–D and I–L).

Despite evidence that demonstrates that NAD^+^ blood levels can increase 2.7-fold after single doses of oral NR [28], many chronic NR studies do not observe effects on biological endpoints until 5–6 weeks on diet [14, 29]. Similarly, we observed a significant main effect of treatment (diet) on time spent in NREMS only after 6 weeks on NR-supplemented diet (NREMS: F_1,14_ = 5.65, *p* = 0.032, Figure 4, E–H). NR supplementation decreased the time that animals spent in NREMS after 6 weeks on diet. These differences are especially clear during lights-on; however, RM ANOVAs for treatment × week and treatment × week × 6-hour interval (i.e. time of day) were not significant. Decreased time spent in NREMS was mirrored by non-significant increases in time spent in wakefulness and REMS. This was partially reflected in a trend towards increased wakefulness (Wake: F_1,14_ = 3.57, *p* = 0.079, Figure 4, A–D).

### NR supplementation does not alter LFP spectral power differences across sleep–wake states

**Figure 5.**
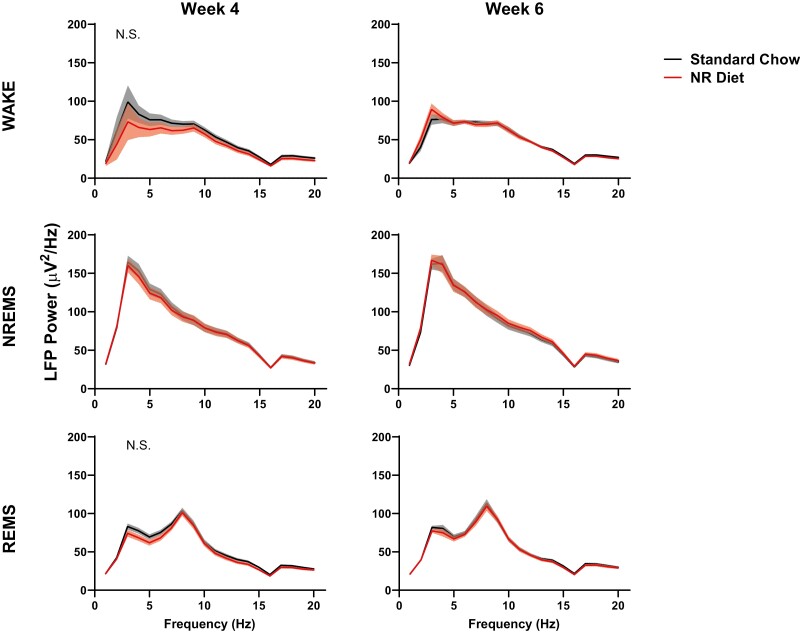
LFP spectral power during wake (A and B), NREMS (C and D), and REMS(E and F) in mice during SS recordings. Spectral data is displayed as the average power across all artifact-free epochs of this state in the 24-hour baseline period. Panels on the left (A, C, E) display mice 4 weeks after starting experimental diets, and panels on the right (B, D, F) display mice 6 weeks after starting experimental diets. Differences in spectral power were assessed between animals on control chow (black/gray) and on NR-supplemented diet (red). Frequency × week × treatment differences were indicated by RM ANOVA, and individual frequency band differences were derived via post hoc assessment by Fisher’s LSD. Differences were only observed at 3 Hz, in wakefulness during week 6. No differences were found between groups in wakefulness at week 4, NREMS at weeks 4 and 6, or REMS at weeks 4 and 6. Significance is indicated by asterisks between these groups where *p* < 0.05. Differences were not modulated by sex. Gray and red shaded areas signify SEM.

To maximize statistical power, we restricted LFP spectral analyses to week 4 versus week 6, when effects of dietary supplementation first emerged. A close examination of daily baseline low-frequency LFP power (1–20 Hz) between weeks 4 and 6 demonstrates very few significant differences aside from increased power in the delta range at week 6 (RM ANOVA week × frequency × treatment interaction; F_19,361_ = 1.90, *p* = 0.013; Figure 5B) during wakefulness. Significance was not detected for week × frequency × treatment or treatment × week across NREM or REM sleep at 4 or 6 weeks on diet. Overall, all curves in Figure 5 demonstrate the expected power distributions during uninterrupted baseline activity.

### SR of NR-supplemented animals does not produce significant differences in sleep timing

We next examined sleep timing during, between, and after 30-minute intervals of SR alternating with 30-minute intervals of undisturbed sleep opportunities (see Figure 2A for SR protocol) to see if the NREM sleep effects observed in Figure 4, during baseline persist with increased sleep pressure. As the effect of NR supplementation on NREM sleep during spontaneous sleep (SS) was observed to be most prevalent during lights-on (ZT0-12), we examined SR (ZT1-7) and subsequent recovery sleep (ZT7-10) during this time of day to eliminate questions of circadian impact.

Time spent in wake, NREMS, and REMS across the six 30-minute intervals of SR were not significantly affected by treatment, treatment × week interaction, or treatment × week × 6-hour interval interaction (all *p* > 0.17; data not shown). Further examination of wake timing during SR intervals shows that the SR protocol maintained efficacy across the 6-week experimental timespan, as mice are awake for a majority of all 30-minute intervals. Differences between NR and control groups in sleep timing did not reach significance after 6 weeks of diet.

Time spent in wake, NREMS, and REMS across the six 30-minute intervals of undisturbed sleep opportunities immediately after each 30-minute SR interval (recovery sleep opportunities) were not significantly affected by treatment, treatment × week interaction or treatment × week × 6-hour interval interaction (all *p* > 0.05; data not shown). Increasing sleep pressure via SR appears to eliminate the significance of the NR-induced NREM sleep reductions observed during undisturbed sleep in baseline recordings after 6 weeks of NR supplementation.

Time spent in wake, NREMS, and REMS across the 3 undisturbed hours of SS recordings occurring after the final 30-minute SR interval were also not significantly affected by treatment, treatment × week interaction, or treatment × week × 6-hour interval interaction (all *p* > 0.11; data not shown).

### Sleep is more efficient with NR diet

**Figure 6.**
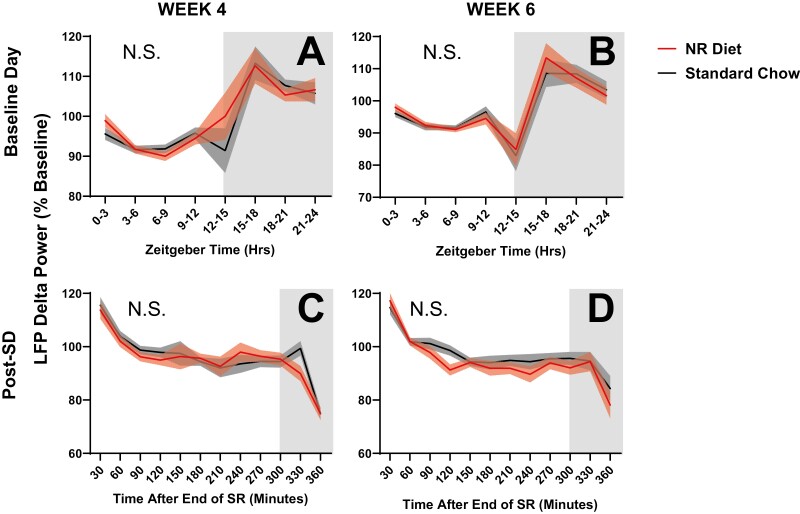
LFP delta power during SWS at 4 weeks (A and B) and 6 weeks (C and D) after starting experimental diets. Data are normalized to the mean delta power across all artifact-free epochs of NREMS in the 24-hour baseline recording. Panels (C and D) additionally show delta power for 6 hours of uninterrupted, spontaneous sleep immediately after the sleep restriction period (starting at ZT7). Differences in spectral power were assessed between animals on control chow (black/gray) and on NR-supplemented diet (red). Week × diet × frequency was assessed in 3-hour (A and B) or 30-minute (C and D) bins. No differences were found between groups and no sex-related differences were observed. Shaded areas signify SEM.

**Figure 7.**
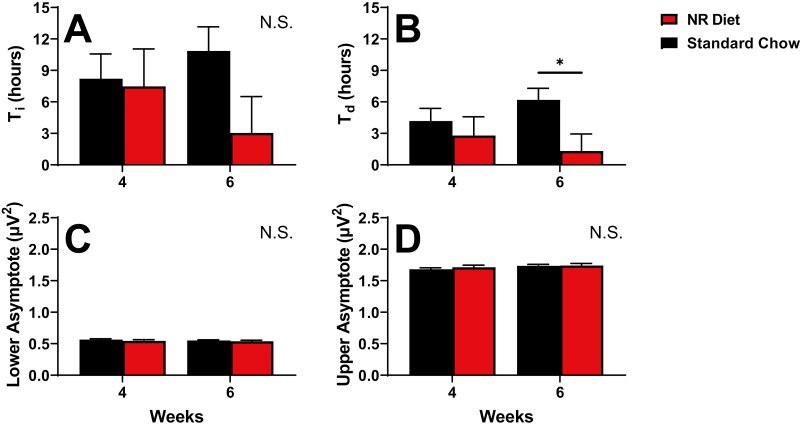
Parameters of Process S 4 and 6 weeks after starting on experimental diets. Four parameters are displayed here, including the rising time constant (A: τ_i_), the falling time constant (B: τ_d_), lower asymptote (C: LA), and upper asymptote (D: UA). Differences were assessed between animals on control chow (black) and NR-supplemented diet (red) by RM ANOVA. Treatment × week interactions were further probed via post hoc assessment by Fisher’s LSD test. Differences were only observed at week 6, in τ_d_ (B). No differences were found between groups in τ_i_, LA, or UA. Significance is indicated by asterisks between these groups where *p* < 0.05. Differences were not modulated by sex. Error bars signify SEM.

Sleep disturbances that parallel aging-related NAD^+^ decline have been demonstrated to manifest as decreases in total sleep time and slow wave sleep (i.e. NREMS) as well as increased time spent awake during the biological night, resulting in reduced sleep efficiency [30]. Our observations of decreased NREM sleep after 6 weeks of NR supplementation (Figure 4, F–H) led us to assess several additional parameters that describe sleep efficiency. These include NREM bout count and bout duration (i.e. sleep consolidation), the rate of EEG/LFP delta power discharge (Figure 6), and parameters of Process S (Figure 7).

No effects of diet were observed on sleep consolidation during baseline, SR, or recovery sleep opportunities. Mean bout durations for wake, NREMS, and REMS were not significantly affected by treatment, treatment × week interaction, or treatment × week × 6-hour interval interactions. The number of bouts of wake, brief awakenings, NREMS, and REMS were also not affected by treatment, treatment × week interaction, or treatment × week × 6-hour interval interactions (data not shown).

To assess the daily discharge of SWA, baseline (SS) normalized delta power during NREMS was compared 4 and 6 weeks after the start of dietary supplementation. Any differences that manifested after NR-supplementation should be apparent when comparing week 4 data to week 6 data (see Figure 4). No significant differences were observed between the delta power values of animals on control chow and those on NR diet at week 4 (Figure 6A) or week 6 (Figure 6B). Because sleep pressure increases as a function of prior wakefulness, we also assessed accelerated delta discharge during the recovery sleep period after SR (starting at ZT7, see Figure 2A). Post-SR delta power was also not affected by diet after 4 and 6 weeks of diet in NR-supplemented animals when compared to those on control chow (Figure 6, C and D). Delta power during the 30-minute SR sessions, as well as 30-minute interspersed sessions of recovery sleep were also not significantly affected by NR supplementation (data not shown).

Finally, we assessed the dynamics of SWA by fitting our data to a homeostatic “Process S” model (Figure 7). Process S quantifies the accumulation of sleep pressure during wakefulness and its discharge during sleep. The specific model used here incorporates all data from each mouse (baseline, SR, and recovery sleep), as previously described [18]. The incorporation of these varied components allows for assessment of the epoch-to-epoch dynamics of wake–sleep, giving us a more granular assessment of sleep pressure than the analysis described in Figure 6. Four of the parameters that define Process S (the rising time constant: τ_i_; the falling time constant: τ_d_; upper asymptote: UA; lower asymptote: LA) are displayed in Figure 7 to describe the sleep of mice on NR-supplemented and control chow at weeks 4 and 6 after starting diet. The UA and LA represent the theoretical upper and lower limits of sleep pressure, respectively. RM ANOVAs assessing treatment and treatment × week interactions for UA and LA were not significant (Figure 7, C and D), indicating that the dynamic range of EEG delta power was not changed by NR-supplementation. At 6 weeks on diet, τ_d_, the time constant for discharge of sleep pressure, was lower in mice on NR-supplemented diet than those on control chow after 6 weeks on diet (Figure 7B; F_1,19_ = 6.2, *p* = 0.022; RM ANOVA, treatment × week;). Note that these effects manifest at week 6, but not at week 4 (Figure 7B). τ_d_ values indicate that chronic NR supplementation (6 + weeks) accelerates the rate at which sleep pressure discharges in mice by more than 4-fold. This could explain the decreased NREM sleep duration seen during SS at baseline (Figure 4, F and H), as well as the trends towards reduced NREM sleep during and after SR (Figure 4, E–H).

#### NR supplementation decreases the cumulative manifestation of sleep need during quiet wake and its discharge during NREM sleep.

**Figure 8.**
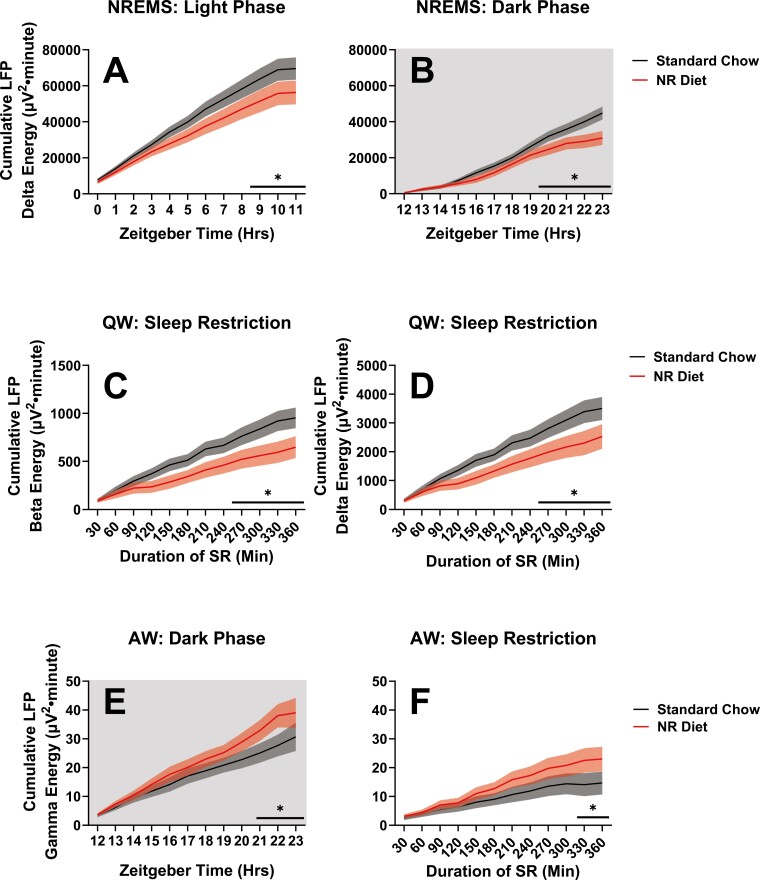
Changes in net slow wave activity between mice on control chow (black/gray) and NR-supplemented diet (red) during NREMS, after 6 weeks on diet. Data are displayed in 1-hour bins. Data are displayed as cumulative delta energy (i.e. average delta power × the number of SWS epochs per hour, added cumulatively across time intervals). Panel A displays cumulative LFP delta energy for mice during the light phase (ZT0-12) during baseline SS recordings. Panel B displays the same for mice during the dark phase (ZT12-23). Treatment × time differences were indicated by RM ANOVA, and interval-specific differences were derived via post hoc assessment of NR versus control diet at each interval by Fisher’s LSD. Significant differences were found at ZT9-11 and 20–23. Panels C and D display changes in cumulative beta energy (C) and cumulative delta energy (D) between mice on control chow (black/gray) and NR-supplemented diet (red) in QW during the SR protocol after 6 weeks on experimental diets. Data are displayed in 30-minute bins, starting at ZT1, and ending at ZT7. The sleep restriction protocol used here alternates periods of experimenter intervention with sessions of recovery, the former allows for periods of wakefulness during SR (see Figure 2A). Treatment × time differences were indicated by RM ANOVA, and interval-specific differences were derived via post hoc assessment of NR versus control diet at each interval by Fisher’s LSD. Significant differences were found from 270 to 360 minutes (i.e. the final 90 minutes of the sleep restriction protocol) in both delta and beta-cumulated energies during QW. Panels (E and F) display changes in cumulative gamma energy between mice on control chow (black/gray) and NR-supplemented diet (red) in AW after 6 weeks on diet. Panel E displays LFP cumulative gamma energy for mice during the dark phase of the baseline SS recordings (ZT12-23, data displayed in 1-hour bins). The panel F displays LFP cumulative gamma energy for mice during the SR protocol described in Figure 2A (ZT1-7, data displayed in 30-min bins). Treatment × time differences were indicated by RM ANOVA, and interval-specific differences were derived via post hoc assessment of NR versus control diet at each interval by Fisher’s LSD. Significant increases in cumulated gamma with NR diet were found in the last 3 hours of the dark phase (A), and the last half hour of SR (B). Significance is indicated by asterisks between these groups where *p* < 0.05. Differences were not modulated by sex. Red and gray shaded areas signify SEM.

Despite nonsignificant changes in delta power between animals on NR-supplemented and control chow during SWS, Process S analysis demonstrated that sleep efficiency was improved after 6 weeks on diet (Figure 6, Figure 7). Current theories of sleep regulation demonstrate that Process S homeostatically regulates delta power over time (i.e. cumulated delta energy), rather than delta power segmented across intervals of time (Figure 6) [31, 32]. Accordingly, assessment of cumulated LFP delta energy across week 6 baseline sleep demonstrated changes in sleep efficiency (Figure 8, A and B). During spontaneous NREM sleep at baseline after 6 weeks of NR supplementation, cumulative delta energy was significantly reduced in the light phase (RM ANOVA Treatment × time interaction; F_11,209_ = 2.46, *p* = 0.007; Figure 8A) and dark phase (RM ANOVA Treatment × time interaction; F_11,209_ = 3.89, *p* < 0.001; Figure 8B), signifying a decreased expression of sleep need.

Whereas delta power tracks the discharge of sleep need during NREMS, delta power during QW tracks the accumulation of sleep need [15]. NR-induced decrements in delta accumulation persisted across QW during SR and the intervening sessions of recovery (RM ANOVA Treatment × time interaction; F_11,209_ = 70.14; *p* < 0.001; Figure 8D).

Prior work from our lab has indicated that beta-band activity also tracks with delta oscillations during QW, as a marker of sleep need [14]. Cumulated QW beta energy of NR-supplemented animals during the SR protocol also demonstrates reductions after 6 weeks on diet (RM ANOVA treatment × time interaction; F_11,209_ = 3.54; *p* < 0.001; Figure 8C). Along with the changes observed in cumulative delta energy (Figure 8, A and B), these data support our Process S analysis (Figure 7), which indicates reduced accumulation of sleep pressure across wakefulness, even when sleep drive is high during SR.

Finally, we further investigated LFP energies during active wake (AW) to better understand the trends towards increased wakefulness in mice receiving chronic NR supplementation (Figure 4, B and D, 6, B and D, 7, B and D, 8, E and F). Cumulated gamma energy in the most active time periods—the dark phase and SR—was found to build at a significantly faster rate after 6 weeks of NR-supplementation (dark phase at baseline: RM ANOVA Treatment × time, F_11,209_ = 75.66, *p* < 0.001, Figure 8E; during SR: RM ANOVA Treatment × time, F_11,209_ = 2.26, *p* = 0.013, Figure 8F). As gamma oscillations are hypothesized to support cortical processes such as attention, learning, memory, and visual processing [33], it is possible that chronic NR supplementation increases the capacity for complex cognitive processes.

Effects of diet on cumulated power were restricted to delta, beta, and gamma ranges and were detected only after 6 weeks of treatment (Figure 8). No significant differences in cumulative LFP energies were detected at week 4 (data not shown).

## Discussion

Here, we describe the effects of systemic manipulation of redox substrates on sleep timing and the electroencephalographic features associated with sleep homeostasis. We hypothesized that increased glucose utilization during prolonged wakefulness (SR) would impact the redox status of the brain due to a prolonged need for oxidative metabolism. We expected that increasing the redox capacity of the cortex via increasing the availability of redox substrates—namely, NAD+/NADH (via NR) would protect against the shift to oxidation caused by SR. We expected this treatment to protect against reductions in gamma activity and increases in delta activity and sleep propensity that occur during sustained intervals of spontaneous or enforced wakefulness [15]. Interestingly, while the impacts of NR concurred with our expectations, the observed effects were more robust during spontaneous, uninterrupted sleep than during SR. NR supplementation impacts cellular redox capacity via the biochemical pathway illustrated in Figure 1. Its effects on sleep timing and EEG measures show that this pathway is capable of modulating variables associated with sleep homeostasis.

### Sleep-related effects of NR are observed after 6 weeks of chronic supplementation

Orally supplemented NR is uniquely bioavailable as an NAD^+^ substrate, with a superior pharmacokinetic profile to nicotinic acid and nicotinamide [28, 34]. Studies of NR show that even single oral doses can increase blood NAD^+^ levels up to 2.7 times. However, blood levels do not immediately correlate with tissue absorption of NR, as maximal gut absorption takes a minimum of 1.5 weeks of supplementation [33]. The short elimination half-life reported in several studies further suggests that NR needs to be taken daily to maintain efficacy with regard to increasing tissue levels of NAD^+^ [35].

A still longer protocol of dietary NR supplementation demonstrated that 6 weeks of NR administration resulted in increased neurogenesis in the brains of C57BL6/J mice [14]. Because this protocol demonstrated a timescale for brain-specific effects, we used it to inform the timeline of our own study. We found that NR produced sleep-related effects, measured via LFP-related parameters after a minimum of 6 weeks of chronic supplementation; assessment at 4 weeks of NR supplementation did not show changes. Although the bioavailability of NR is better than other NAD^+^ precursors, its low tissue absorption and rapid degradation into other metabolites such as nicotinamide likely delay the saturation of NAD^+^ in cortical tissue [35]. Additionally, NAD declines with aging [36]. It is possible that the brief time window of aging assessed here (i.e. 10–20 weeks of age) produces sufficient decreases in endogenous NAD^+^ such that effects of NR supplementation on sleep are observed, and that these effects are only significant after the mice are 16 + weeks old. As previous studies have demonstrated that the magnitude of NR’s blood-level saturation are greatest in individuals with naturally low blood NAD^+^ levels [37], changes observed past 16 weeks of age may be attributable to NAD^+^ saturation reaching equilibrium after an initial spike in response. Since we did not measure tissue-level NAD^+^ concentrations in this report, these possibilities are all hypothetical.

While Zhang et al. demonstrated neurogenesis using the same protocol, they do not describe any sleep-related effects [14]. Very few studies have assessed effects of NR on sleep–wake cycles and sleep–wake EEG parameters. Only two other studies have reported effects on sleep–wake cycles to date, both of which required a minimum of 6 weeks of NR supplementation before effects were observed [22, 25].

Although many studies use chronic NR supplementation, few have taken multiple assessments of biological endpoints over the course of administration. Those which have only reported assessments of the pharmacokinetic profile of NR during nutritional supplementation [34]. Our study is unique in that it assesses our biological endpoints multiple times throughout the course of the study, ensuring a clear understanding of the time course of our results. Sleep and electroencephalographic results were achieved at 6 weeks of NR supplementation and maintained for at least 1 month afterward (up to 10 weeks on diet).

### Effects of NR supplementation are different during uninterrupted sleep, spontaneous sleep, and SR

We expected that SR would exacerbate deficits in reduced NAD (NADH) produced by prolonged wakefulness. Because waking waveforms require increased energy and oxidative metabolism, disruptions of sleep generally demonstrate increases in oxidative stress [7, 38, 39]. We hypothesized that increasing the availability of redox substrates (i.e. NAD) via NR supplementation would decrease sleep drive. By this logic, we expected the effects of NR supplementation to be greatest when sleep drive was greatest (during SR), allowing NR to compensate for the effects of SR. However, our results demonstrate that NR supplementation produces larger decrements in time spent in spontaneous NREM sleep during SS (Figure 4), than it does during or after SR (Figures 6–8). Additionally, while NR-supplementation produces a slower rate of sleep drive accumulation during SR (Figure 8D), delta oscillations decline at the same rate in animals fed control chow and NR-supplemented diet during recovery sleep. This can be contrasted with the effects of NR-supplementation on sleep drive during SS; in NR-supplemented animals, sleep drive accumulates normally during SS but is discharged at a faster rate (Figure 7).

### NR supplementation modifies the sleep homeostat

The state-specific dynamics (Figure 5) and homeostatic dynamics of LFP power within SWS (Figure 6) demonstrate that NR-supplemented animals maintain the ability to generate delta oscillations in response to prior sleep–wake history. However, NR modifies the gain of the sleep homeostat. The “gain” of a homeostatic system defines the efficiency with which it is regulated. Gain describes the rate of a driven variable’s change in response to a changing driving variable. In the sleep homeostat, SWS delta power is driven to change by prior time spent in NREM sleep (i.e. sleep–wake history). The rate at which delta power changes during SWS is measured by τ_d_, the declining time constant, which quantifies the rate of discharge of sleep need. NR supplementation decreases τ_d_, indicating increased gain (i.e. increased rate of delta discharge). τ_i_ is the driving variable for the increase in delta power as a function of time spent awake. While τ_i_ was not significantly affected in the Process S model albeit with a trend toward reduced τ_i_ with NR supplementation, this observation further demonstrates the ability of NR to modify dynamics of Process S by increasing the availability of substrate for NAD-dependent redox reactions (Figure 7).

Of the numerous NAD-dependent enzymatic processes that exist, many could underlie the observed alterations to the sleep homeostasis. Two NAD-dependent enzymes known to contribute to sleep regulation are potential candidates that might underlie effects on Process S: Poly (ADP-ribose) polymerases (PARPs) [40] and sirtuin 1 (SIRT1) [41].

### Changes in gain are exemplified by measurements of LFP energy

LFP energy is the time integral of power, meaning that it quantifies cumulative changes in power over time. When assessing LFP energy, we observed the effects of NR as described by Figure 7: decreased delta energy during NREMS in SS (Figure 8, A and B), decreased delta energy accumulation during QW in SR (Figure 8, C and D), and increased gamma energy during AW in SS and SR (Figure 8, E and F). Importantly, these results align with findings in the literature regarding sleep and NR. In a human study, Remie et al. demonstrated an increased sleeping metabolic rate after 6 weeks of NR supplementation [22]. These increases in sleeping metabolic rate can support higher energy oscillations during NREMS. Delta oscillations are associated with decreases in cerebral lactate concentration [15], although this decrease may be related to increased glymphatic clearance of lactate [42] in addition to reduced glycolytic production of lactate [1–4],. Accordingly, our study demonstrates that NR-supplementation decreases the time spent in NREMS undergoing low-energy delta oscillations (during SS; Figure 8, A and B).

Finally, a recent study demonstrated use of NR to restore circadian rhythmicity of locomotor activity cycles in aged mice. Aging produces dampening of locomotor activity cycles regulated by process C, the circadian process of the two-process model. This creates issues such as increased daytime napping and shifts toward earlier wake times and sleep onset. In that study, NR supplementation restored SIRT1 activity, BMAL1/chromatin binding, as well as mitochondrial respiration rhythms, resulting in restored rodent activity in the late evening [25]. Our work complements these conclusions, as we show increases in wakefulness with NR. In future studies, we aim to measure Process C (by studying animals in constant dark conditions) as well as Process S to further replicate these recent findings.

The congruence between our results presented here and those presented in the literature further validates the robust effects of NR supplementation on sleep. Many other NAD manipulations along the NAD^+^ salvage pathway provide less robust data, and subsequently do not provide a clear picture in the literature. For example, sleep-related work with nicotinamide mononucleotide (NMN) supplementation demonstrates reduced drowsiness or no effects on sleep, depending on the study [26, 43]. This is possibly due to the low bioavailability and varying efficacy of NMN treatments.

### Translational relevance of the findings

The effects of NR on sleep are likely to have translational relevance, as there is considerable evidence that dietary nicotinamide impacts brain chemistry and function in human participants [44–47]. NR supplementation (30 days @ 1 gram NR per day) in Parkinson’s disease patients resulted in an increase in brain nicotinamide concentration of approximately 30%, as assessed by ^31^P-magnetic resonance spectroscopy [12]. Cerebrospinal fluid concentration of N-methyl-2-pyridone-5-carboxamide, a metabolite of nicotinamides, was elevated in the same patient population [12]. Functional endpoints related to disease outcome were not reported from this ongoing clinical trial, though previous studies suggest that NR supplementation is of therapeutic benefit in Parkinson’s disease [46]. Collectively, the human magnetic resonance spectroscopy data, the biochemical data from human cerebrospinal fluid [12] and rodent brain tissue [48], and the efficacy of nicotinamide supplements in human neurological conditions demonstrate feasibility of dietary enhancement of brain nicotinamide as a therapeutic strategy in humans.

Our data demonstrate that dietary enhancement of brain nicotinamide curtails sleep by accelerating the discharge of homeostatic sleep need. Application of dietary nicotinamide supplements to humans is highly feasible in the immediate future. Dietary nicotinamide supplements are not an FDA-regulated pharmaceuticals; they are considered a dietary supplement in the United States. Dietary nicotinamide supplements have been used in hundreds of completed and ongoing, non-sleep-related, clinical trials registered with clinicaltrials.gov (of which one trial measures sleep, but only through self-reported sleep quality). It would be of considerable translational significance to replicate our finding of reduced sleep need in a human study and to demonstrate the effectiveness of dietary nicotinamide supplementation in simulated operational settings.

NAD (as a coenzyme) aids cellular enzymatic activity, which includes DNA repair, cellular metabolism, and cell stress responses. Perturbation of NAD^+^ and downstream signals mediated by SIRT1 due to sleep deprivation have been linked to neurodegenerative processes [41]. While the current report indicates reduced time spent asleep under NR diet, it is unclear whether NR diet mitigates against SR-induced cellular insults, as they were not measured in the current study.

### Current limitations

While the study described here provides new insight into the effects of nicotinamide supplementation on sleep and its underlying EEG manifestations, several limitations of the design must be acknowledged. First, the SRs described in these studies started at ZT1 rather than at the time of light onset (ZT0), which may have blunted the homeostatic response to SR.

Secondly, these studies do not include metabolic endpoints or methods, which quantify tissue-level oxidative stress and antioxidant capacity before, during, or after the treatments assessed. While we can correlate our findings with previous observations from the literature on studies that manipulate nicotinamide concentrations, we cannot make direct conclusions regarding the effects of our treatments on cellular metabolism. Future studies would benefit from coupling EEG and sleep-related assessments with further cellular and metabolic assessments from the brain and other tissues that are known to be impacted by dietary NR [20–23]. Longitudinal measurements taken from metabolic chambers would be particularly useful in understanding how metabolic changes might contribute to sleep-related effects of these treatments.

Lastly, the generalizability of the results may be limited by the model organism in which studies were performed. C56BL/6J mice have a mutation in the nicotinamide nucleotide transhydrogenase (NNT) gene, which encodes an enzyme that serves to reduce NADP^+^ to NADPH at the expense of NADH oxidation and H^+^ reentry to the mitochondrial matrix via the inner mitochondrial membrane. In doing so, it provides a major pathway by which NADP^+^/H and NAD^+^/H interconvert within the cell. The mutation carried in C57BL/6J mice results in mitochondrial redox abnormalities [49]. Effects of NNT deficiency include: higher rates of hydrogen peroxide release and poorer ability to metabolize peroxide; spontaneous NADPH oxidation; and increased ratio of oxidized glutathione to reduced glutathione. Net effects are increased oxidative stress and blunted glutathione-based antioxidant capacity in C57BL/6J mice [49]. These phenotypes may make C57BL/6J more sensitive to redox perturbations than other mice, and thus potentially more responsive to NR.

The current experiment aimed to replicate the timeline of neurological effects seen in C57BL/6J mice after NR-supplementation in prior studies [14], and thus applied the same strain of mice. Other supplements that target glutathione-dependent antioxidant pathways (e.g. N-acetylcysteine) have also demonstrated neuroprotective effects in C57BL/6J mice in the literature [50–52]. It is likely that redundancies in nicotinamide biochemistry—about 200 enzymes process nicotinamide-based substrates- allow for efficient use of nicotinamides despite the NNT mutation. However, a future study would do well to assess whether the effects of NR on EEG and sleep differ between C57BL/6J and other strains with intact nicotinamide pathways (such as C57BL/6N mice).

## Data Availability

The data underlying this article will be shared on reasonable request to the corresponding author.
